# Impulsivities and Parkinson's Disease: Delay Aversion Is Not Worsened by Deep Brain Stimulation of the Subthalamic Nucleus

**DOI:** 10.1371/journal.pone.0043261

**Published:** 2012-09-11

**Authors:** Diana M. E. Torta, Vincenzo Vizzari, Lorys Castelli, Maurizio Zibetti, Michele Lanotte, Leonardo Lopiano, Giuliano Geminiani

**Affiliations:** 1 Department of Psychology, University of Turin, Turin, Italy; 2 Department of Neuroscience, University of Turin, Turin, Italy; University of Medicine & Dentistry of NJ - New Jersey Medical School, United States of America

## Abstract

Deep Brain Stimulation (DBS) of the Subthalamic Nucleus (STN) improves motor symptoms in Parkinson's disease (PD), but can exert detrimental effects on impulsivity. These effects are especially related to the inability to slow down when high-conflict choices have to be made. However, the influence that DBS has on delay aversion is still under-investigated. Here, we tested a group of 21 PD patients on and off stimulation (off medication) by using the Cambridge Gamble Task (CGT), a computerized task that allows the investigation of risk-related behaviours and delay aversion, and psychological questionnaires such as the Barratt Impulsiveness Scale (BIS), the Sensitivity to Punishment and to Reward Questionnaire (SPSRQ), and the Quick Delay Questionnaire (QDQ). We found that delay aversion scores on the CGT were no higher when patients were on stimulation as compared to when they were off stimulation. In contrast, PD patients reported feeling more impulsive in the off stimulation state, as revealed by significantly higher scores on the BIS. Higher scores on the sensitivity to punishment subscale of the SPSRQ highlighted that possible punishments influence patients' behaviours more than possible rewards. Significant correlations between delay aversion scores on the CGT and QDQ delay aversion subscale suggest that these two instruments can be used in synergy to reach a convergent validity. In conclusion, our results show that not all impulsivities are detrimentally affected by DBS of the STN and that the joint use of experimental paradigms and psychological questionnaires can provide useful insights in the study of impulsivity.

## Introduction

Deep Brain Stimulation (DBS) of the Subthalamic Nucleus (STN) represents a therapeutic advance for severely disabled patients with Parkinson's disease (PD) [1], [2], [3], [4], [5]; it inhibits hyper-activation of the STN, therefore acting as a reversible lesion of the target areas. STN-DBS is efficacious on motor symptoms [1], [2], [3], [4] and enables the reduction of dopaminergic treatment [6]. Moreover, DBS can be considered globally safe in terms of cognitive outcomes both in the short [1], [7], [8] and long term [6], [9], [10], even though single patients may develop a clinically relevant cognitive decline [11]. Although some investigators have shown that STN-DBS can reduce impulsive behaviours [12], [13], [14], [15], [16], [17], others have reported detrimental effects of DBS [15], [18], [19], see [20] for a review. Risk factors for the development of post-surgery impulsive behaviours include premorbid susceptibility [20], but the exacerbation of an impulsive symptomatology may also derive from the progress of the pathology and from DBS itself [21], [22]. Studies conducted with the use of self-administered questionnaires, such as the Baratt Impulsiveness Scale (BIS), have shown that STN-DBS treated patients feel more impulsive than patients receiving only medical therapy [18]. However, self-administered questionnaires may be susceptible to subjective over- or under-estimations of the severity of the symptomatology [23], [24]. Thus the results of such questionnaires, where possible, should always be compared to those of experimental paradigms to reach a convergent validity. Experimental paradigms can provide greater sensitivity to study impulsivity [22]. For instance, it has been documented that, as compared to the off stimulation condition, PD patients on stimulation have a poorer performance in go/no-go tasks [25], [26], [27], stop signal tasks [28] and fail to slow down in selecting responses in a high-conflict context [29], [30], [31]. Importantly, the concept of impulsivity is not unitary - indeed, impulsive behaviours reflect the inability to use externally available information to reflect and ponder future actions and their consequences (cognitive impulsivity); the inability to opt for larger, delayed rewards rather than smaller, immediate ones (delay aversion), and impairment in suppressing prepotent motor responses (motor impulsivity/impulsive action) [32]. These different aspects of impulsivity are dissociable at both the neuroanatomical and neuropharmacological levels [33], [34]. Preclinical models have demonstrated that lesions of the STN have opposing behavioural effects on different aspects of impulsivity (see [33]). Although the effects of STN-DBS on cognitive and motor impulsivity have been explored [25], [26], [29], [31], [35], experimental evidence on the effects of DBS on delay aversion is still lacking. Interestingly, preclinical studies suggest that lesions to the STN may even improve delay aversion in rats [36].

Here, we first investigated the effect of STN-DBS on delay aversion in patients on stable DBS stimulation by using the Cambridge Gamble Task (CGT) [37], a computerized task for the assessment of delay aversion, and psychological questionnaires. We then investigated the possible short-term effects of DBS on self-reported measures of impulsivity, comparing psychological questionnaire scores between the off and on stimulation conditions.

## Materials and Methods

### Patients

This study was approved by the Department of Psychology, University of Turin Ethical Committee and all participants gave their written informed consent. Twenty-one PD patients participated in this study (8 women). Of these, thirteen completed the entire battery composed of the CGT [37] and the psychological questionnaires (Barratt Impulsiveness Scale, BIS, Italian version [38], the Sensitivity to Reward and Sensitivity to Punishment Questionnaire, SRSPQ [39], the Quick Delay Questionnaire, QDQ [40], see next paragraphs), while eight completed only the CGT. The patients had an average age of 60 (±6.2, range 48–70) and an average 8.6 years of schooling (±3.8, range 5–17). They were evaluated at an average of 9.5 months (±4.9, range 4–28) after implantation of electrodes for DBS of the STN. This allowed us to ensure that patients were on stable stimulation and no longer prone to microlesive effects of the surgical procedure [10]. A post-operative 3D CT fused with the pre-operative MRI scan was performed to confirm successful surgery and to check the final position of the electrodes. The patients were administered psychological and neuropsychological assessments as clinical routine when on stimulation and on medication. These assessments were used to exclude clinically relevant psychological symptoms and neuropsychological impairments [11]. In addition to dopaminergic therapy, seven patients were taking antidepressants (six were on serotonin-norepinephrine reuptake inhibitors (SNRIs) and one on a selective serotonin reuptake inhibitor (SSRI), six on benzodiazepines, five on atypical antipsychotics). Table 1 summarizes the clinical and demographic details.

**Table 1:** Clinical and demographic characteristics of the PD sample.

Patient code	Gender	Age	Years of schooling	Months after surgery	UPDRS III on/off	LEDD mg/day	Parameters of stimulation right/left
1	F	59	17	12	24/33	1600	3.6 V; 60 µsec; 130 Hz; 11-/3.4 V; 60 µsec; 130 Hz; 3-
2	F	60	8	11	5.5/14	505	3.4 V; 60 µsec; 130 Hz; 10-/3.3 V; 60 µsec; 130 Hz; 2-
3	F	57	9	11	23/35	580.19	3.6 V; 60 µsec; 130 Hz; 11-/3.6 V; 60 µsec; 130 Hz; 3-
4	F	48	11	10	29/59	500	2.4 V; 60 µsec; 130 Hz; 10-/2.4 V; 60 µsec; 130 Hz; 1-
5	M	57	11	10	22/45.5	1180.19	3.6 V; 60 µsec; 130 Hz; 8-/3.6 V; 60 µsec; 130 Hz; 0-
6	F	69	5	5	15.5/22.5	771.58	3.6 V; 60 µsec; 130 Hz; 10-/3.3 V; 60 µsec; 130 Hz; 2-
7	M	57	10	5	23.5/43.5	750.38	3.6 V; 60 µsec; 130 Hz; 9-/3.6 V; 60 µsec; 130 Hz; 1-
8	M	59	5	10	36.5/49.5	500	3.6 V; 60 µsec; 130 Hz: 10-/3.0 V; 60 µsec; 130 Hz; 1-
9	F	60	10	7	30.5/47	625	3.0 V; 60 µsec; 130 Hz; 11-/3.5 V; 60 µsec; 130 Hz; 3-
10	M	64	5	4	7/36	1310	2.8 V; 60 µsec; 130 Hz; 3-/3.2 V; 60 µsec; 130 Hz; 7-
11	M	49	8	7	31/65	1200	3.5 V; 60 µsec;130 Hz; 7-/3.2 V; 60 µsec;130 Hz; 1-
12	M	58	5	6	46/73	554	3.2 V; 60 µsec;130 Hz; 3-/3.2 V; 60 µsec;130 Hz; 7-
13	M	67	5	11	13.5/31.5	600	3.2 V; 60 µsec; 130 Hz; 4-/3 V; 60 µsec;130 Hz; 1-
14	F	67	5	3	21/56.5	525.19	2.9 V; 60 µsec; 130 Hz; 6-/2.7 V; 60 µsec; 130 Hz; 2-
15	M	70	5	17	18.5/39.5	350.19	2.9 V; 60 µsec; 130 Hz; 5-/3.0 V; 60 µsec; 130 Hz; 2-
16	M	62	13	9	12/48	910	5 V; 60 µsec; 80 Hz; 5-/5 V; 60 µsec; 80 Hz; 1-
17	F	71	12	11	30.5/48	700.19	3.4 V; 60 µsec; 130 Hz; 7-/3.2 V; 60 µsec; 130 Hz; 2-
18	M	50	5	30	22.5/42	187.5	2.8 V; 60 µsec; 130 Hz; 7-/3.2 V; 60 µsec; 130 Hz; 3-
19	M	60	8	10	46/70	475.19	3.6 V; 60 µsec; 130 Hz; 2-/3.4 V; 60 µsec; 130 Hz; 7-
20	M	55	15	6	17/37.5	835	3.5 V; 60 µsec; 130 Hz; 7-/3.5 V; 60 µsec; 130 Hz; 3-
21	M	65	5	5	23.5/55.5	650	3.4 V; 60 µsec; 185 Hz; 3-/3.6 V; 60 µsec; 185 Hz; 6-

LEDD, Levodopa Equivalent Daily Dose.

### Experimental procedure

All patients performed the tasks after at least 12 hours (overnight withdrawal) of wash-out from the habitual dopaminergic therapy (off medication). The patients performed the experimental procedure twice - ‘on stimulation’ and ‘off stimulation’ - and the order of administration (with the first experimental session either on or off stimulation) was counterbalanced across subjects. The ‘off stimulation’ session was begun at least 40 minutes after DBS was switched off. Before the experimental procedure was begun, a neurologist administered the UPDRS-III to ascertain that patients were in their off condition. The ‘on stimulation’ session, when not the initial one, was begun at least 40 minutes after stimulation was switched on. Also in this case, the on condition was tested by the administration of the UDPRS-III. The patients performed the task either on two separate days (N = 3), or on the same day (one session in the morning, another in the afternoon (N = 18)). Preliminary analyses revealed that the results obtained when the task was administered on two separate days were no different from those obtained when it was administered on the same day (p = 0.233). The two groups were therefore analyzed together. In each session, the patients performed the CGT and completed the psychological assessment, which included the BIS, SPSRQ and QDQ. For the psychological questionnaires, they were explicitly asked to respond according to how they felt in the condition of stimulation that was being tested.

### Cambridge Gamble Task

We used the Cambridge Gamble Task [37] to assess delay aversion. In this task, participants are asked to choose whether a yellow token is hidden under one of the ten blue or red boxes arrayed on the top of the screen. The ratio of blue to red boxes varies from 1 blue/9 red to 9 blue/1 red, thus covering all possible combinations; the patients were told that the aim of the game was to increase their points as much as possible. The first choice, regarding which colour to bet on, reflects quality of decision, namely the tendency to select most likely outcomes. The time employed to select the most likely outcome reflects the deliberation time. On this first choice, patients were asked to bet their points with the goal of increasing them (more rational choices are related to most likely outcomes of winning). The amount of points they could bet appeared on the right-hand side of the screen after the first selection was made. The patients placed their bets under two different conditions: ascending and descending. With the former, the points to bet begin with low stakes and increase, whereas with the latter, the points start from high stakes and progressively decrease. The points represent percentages of the total score, but the patients are not explicitly informed of this. The participants are invited to select a bet according to their confidence in the red/blue box decision. An optimal strategy requires modulation of the betting strategy that depends on the chances of winning and is measured by risk adjustment. Delay aversion is defined as the tendency to select always the first bets, in both ascending and descending conditions. The higher the score, the higher the tendency to impulsivity.

### Barratt Impulsiveness Scale

The Barratt Impulsiveness Scale (BIS-11 Italian version, [38]) is a 30-item self-administered questionnaire that measures different aspects of impulsivity (e.g. motor impulsiveness, lack of planning, and attentional impulsiveness). For each statement participants have to indicate how often they think or behave as such (never/rarely, sometimes, often, always). The BIS returns a global score of impulsivity ranging from 30 to 120, the sum of three separate indexes: ‘motor impulsiveness’, MI, with a score range from 11–44 points, ‘non-planning impulsiveness’, NPI, also with a score range from 11–44 points and ‘attentional impulsiveness’, AI, with a score range from 8–32 points. Higher scores indicate greater impulsivity.

### Sensitivity to Reward and Sensitivity to Punishment Questionnaire

The Sensitivity to Reward and Sensitivity to Punishment Questionnaire (SPSRQ) [39] is a 48-item yes/no self-report questionnaire. The SPSRQ is composed of two subscales: Sensitivity to Punishment (SP) and Sensitivity to Reward (SR). These reflect two behavioral systems: inhibition and approach [41], [42]. The Behavioral Inhibition System (BIS) is sensitive to punishment and novel stimuli. It motivates the inhibition of the performance of behaviors that can lead to future punishments and represents a sort of ‘stop’ system, promoting avoidance behaviors. In contrast, the Behavioral Approach System (BAS) represents the ‘go’ signal towards positive reinforcers and rewards. People with a highly activated BAS tend to impulsivity. The score of each subscale varies from 0–24, with higher scores indicating a higher sensitivity to punishment or reward.

### Quick Delay Questionnaire

The Quick Delay Questionnaire (QDQ, [40]) is a 10-item self-report questionnaire in which subjects have to declare their degree of agreement with each statement on a five-point Likert scale. The QDQ is composed of two subscales: delay aversion and delay discounting. Higher scores on the delay aversion subscale indicate the presence of positive emotions during the delay, whereas higher scores on the delay discounting subscale indicate the tendency to make choices leading to long-term high benefits. Both subscales have scores ranging from 5–25 points.

### Statistical analysis

Statistical analyses were performed using the software PASW Statistics 18 (SPSS Inc.).

Data from the ascending and descending conditions of the CGT were averaged together and analyses performed using a paired sample t-test (2-tailed) with a significance threshold of p<0.05. Risk adjustment scores, which include the evaluation of the betting strategy in four conditions of stimulation (6∶4, 7∶3, 8∶2, 9∶1) were analyzed using a repeated-measure ANOVA with the ‘stimulation’ (2 levels, on and off) and ‘bet conditions’ (4 levels, 6∶4, 7∶3, 8∶2, 9∶1) as factors. The Hyundt-Feldt correction was used in case of violation of sphericity [43].

BIS total scores were compared using a paired comparison t-test (2-tailed). Additional comparisons were performed on each subscale separately as maximum scores changed from one subscale to the other. SPSR and QDQ scores were analyzed using a repeated measure ANOVA, using as factors the ‘stimulation’ (2 levels, on and off) and the ‘scale’ (SPSRQ 2 levels, sensitivity to punishment and sensitivity to reward; QDQ 2 levels, delay aversion and delay discounting). These analyses were possible since the subscales of these questionnaires have the same score range.

The psychological questionnaire scores were correlated with CGT scores to investigate whether a convergent validity exists between psychological self-reports and experimental measures. The correlations were performed separately for results in the on and off stimulation conditions.

## Results

Since the results of the CGT did not change whether 13 or 21 patients were included in the analyses, the results of the entire sample are provided.

### Cambridge Gamble Task

**Figure 1:**
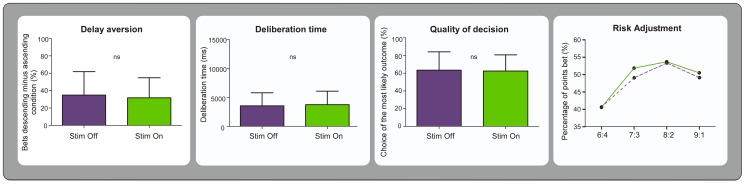
Summary of results on the Cambridge Gamble Task (CGT). Stimulation did not affect the performance in any of the variables of interest. The purple bars and dashed line summarize the results in the off stimulation condition; the green bars and solid line those in the on stimulation condition.

Delay aversion scores were no different in the ‘on’ and ‘off’ stimulation conditions (t(20) = 0.256, p = 0.800). Similarly, the quality of decision and deliberation times were not affected by stimulation (quality of decision, on vs off t(20) = −0.253, p = 0.803; deliberation times, on vs off, t(20) = 0.488, p = 0.631). The analysis of the scores of risk adjustment indicated that patients placed higher bets with more favourable ratios (main effect of ‘bet condition’ F(2,28) = 3.615, p = 0.037), but that these choices were not affected by the stimulation (main effect of ‘stimulation’ F(1,13) = 0.451, p = 0.514). See figure 1.

### Barratt Impulsiveness Scale

The patients felt globally less impulsive when on stimulation (t(12) = 2.680, p = 0.020). They also reported lower scores on the AI subscale when stimulation was on (t(12) = 2.127, p = 0.055). No differences emerged between the MI (t(12) = 1.171, p = 0.264) and NPI (t(12) = 1.367, p = 0.197) subscales in the two stimulation conditions.

### Sensitivity to Punishment and Sensitivity to Reward Questionnaire

The total scores on the SPSRQ were no different in the two stimulation conditions (F(1,12) = 1.581, p = 0.233). However, both when on and off stimulation, patients reported higher scores on the SP subscale (F(1,12) = 6.858, p = 0.022).

### Quick Delay Questionnaire

The total scores on the QDQ were no different in the two stimulation conditions (F(1,12) = 1.990, p = 0.184). No differences emerged in any of the subscales (DA vs DD F(1,12) = 0.073, p = 0.792).

**Figure 2:**
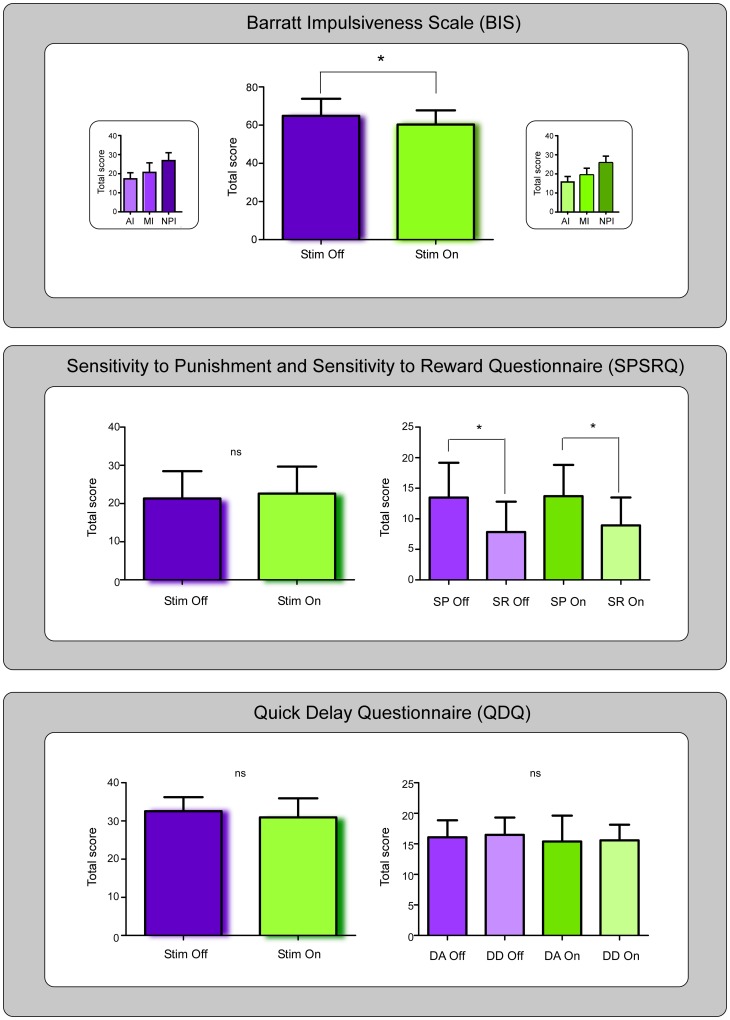
Results on the psychological questionnaires. *Upper panel*: Patients reported lower scores of impulsivity when on stimulation. AI, attentional impulsivity; MI, motor impulsivity; NPI, non-planning impulsivity. *Middle panel:* patients obtained higher scores of sensitivity to punishment than to reward independently of the condition of stimulation. SP, sensitivity to punishment; SR, sensitivity to reward. *Lower panel:* No differences emerged on the QDQ; DA, delay aversion; DD, delay discounting. Please note that unlike the previous two questionnaires, higher scores on the QDQ reflect a lower tendency to impulsivity.

The results are summarized in figure 2.

### Correlations between CGT and questionnaires scores

**Figure 3:**
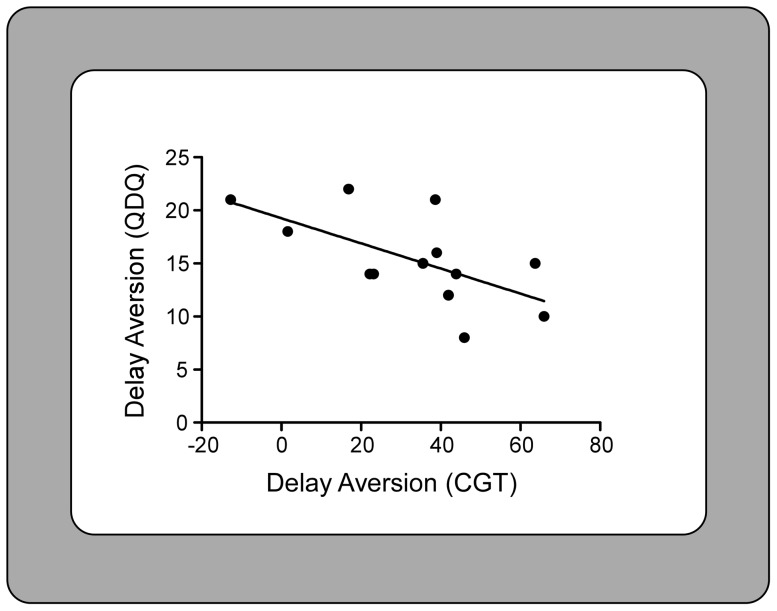
Correlation of delay aversion scores on the Cambridge Gamble Task (CGT) and delay aversion scores on the Quick Delay Questionnaire (QDQ) in the on stimulation condition. A significant negative correlation was found (r = −0.626 p = 0.029) suggesting that higher scores of delay aversion on the CGT were correlated to more negative feelings when waiting for rewards.

A significant negative correlation was found, in the on stimulation condition, between delay aversion scores on the CGT and delay aversion scores on the delay aversion subscale of the QDQ (r = −0.626 p = 0.029). That is, higher scores of delay aversion were correlated to more negative feelings when waiting for rewards (see figure 3).

## Discussion

In the present study we aimed (i) to investigate the effects that STN-DBS has on delay aversion in PD patients and (ii) to explore possible short-term effects of STN-DBS on self-reported impulsivity. We observed two main findings: first, STN-DBS does not worsen delay aversion; second, STN-DBS is able to induce changes in the self-reported psychological state in the short term, that is when switching on and off stimulation.

### ‘Impulsivities’ and Deep Brain Stimulation

Previous animal studies [44], [45] have suggested that, in the rat, lesions to the STN have opposite effects on behaviour: increasing impulsive actions but reducing delay aversion [33]. So far, most of the studies investigating motor (and cognitive) impulsivity in PD patients have found that STN-DBS has detrimental effects on impulsivity as measured by go/no-go tasks [25], [46]. These detrimental effects were found to be particularly related to stimulation of the ventral subthalamic nucleus [47] and accompanied by changes in synaptic activity consisting in reduced activation in the cortical networks responsible for reactive and proactive response inhibition [25]. Moreover, PD patients under STN-DBS have been found to increase impulsive action [48]: STN-DBS, acting detrimentally on the ‘hyperdirect’ circuit connecting the medial prefrontal cortex (mPF) and the STN [49], [50], [51], disrupts the ability to refrain from selecting choices without pondering (DBS). Indeed, STN stimulation was found to diminish theta band power measured by electroencephalography (EEG) in the mPF, which in turn was related to an adaptive increase in response times when the decision process required responses to high-conflict choices [30]. A recent study by Rodriguez-Oroz and colleagues [52] has suggested that patients showing dopaminergic side effects have, in the on state, a characteristic oscillatory activity in the theta-alpha band at different frequencies and with different topography if they suffer from motor (such as dyskinesias) or behavioural side effects (abnormal impulsivity). In contrast, such oscillatory activity does not appear in patients without dopaminergic complications.

Our present findings suggest that STN-DBS does not worsen delay aversion and demonstrate that also in humans not all impulsivities are detrimentally affected by DBS of the STN. These findings, together with the results of previous studies [53], [54], also point to the possibility that some aspects of impulsivity are more affected by dopaminergic therapy than they are by STN-DBS. In this regard, dissociations between the effects of medication and stimulation have been previously documented [48], [55]. In a previous study we reported a positive linear relationship between dopaminergic dose and degree of delay aversion [54], with patients taking higher doses of dopaminergic medication showing higher delay aversion scores. Thus it is likely that when dopaminergic doses are reduced after surgery, the tendency to delay aversion is reduced. According to this explanation, delay aversion is more likely to arise from a dopaminergic overdose of the ventral striatum circuit (overdose theory) [56], [57]. Housden and colleagues [58] reported a double dissociation between reward learning and delay aversion in PD patients with and without impulsive-compulsive disorders, thus proposing that the preference for immediate rewards against future ones is more likely to be underpinned by excessive dopaminergic transmission. An alternative explanation proposed to explain the differences between betting behaviours in the on and off medication conditions states that delay aversion as measured by the GCT reflects ‘impatience’, that is, the inability to undergo delay without action [22]. In this view, impatience could be favoured by different perception of time in the two therapeutic conditions, and in particular by a perception of time going by more slowly in the on medication condition [55], [59], [60], [61]. The possibility that delay aversion, as measured by the CGT, reflects impatience is supported by the negative correlation found between delay aversion on the CGT and delay aversion scores on the DA subscale of the QDQ. Indeed, higher DA scores indicate the presence of positive feelings during delay. None of the other CGT variables were significantly different between the on and off stimulation states, as reported in previous studies [53], [54], thus indicating preserved rational decision making.

### Short-term modification of self-reported impulsivity

The results of the questionnaires indicated that STN-DBS is able to affect the perception of some aspects of impulsivity in the short term. Indeed, patients' reports on the BIS show that they feel more impulsive in the off stimulation condition, especially with regard to attentional impulsivity. These findings are in apparent contrast with previous studies suggesting that PD patients on stimulation report higher scores of self-assessed impulsivity [18]. However, Hälbig and colleagues did not compare scores on the BIS within the same group of patients, but between one group of patients on stimulation and another group on medication.

The results of the SPSRQ revealed a significant difference between SP and SR scores that were independent of the condition of stimulation, that is, patients described themselves as more sensitive to punishment than to rewards. Of note is that PD patients were off medication and that previous studies [62] have shown that unmedicated PD patients are more sensitive to negative than to positive feedbacks in procedural learning tasks. Our results suggest that greater sensitivity to punishment may be a general characteristic of the off medication state, not necessarily confined to procedural learning tasks [62]. This interpretation is further supported by the fact that, although this is the first study using the SPSRQ to test Parkinson patients and therefore no specific normative data are available for such population, in the original article by Torrubia and colleagues [39], the SR and SP scores appear to be very similar (SR: 11.98 (5.06) for women and 11.65 (5.27) for men, SP: 10.11 (4.05) for women and 12.18 (4.48) for men), thus suggesting that, in control subjects, the two systems are balanced.

Not all previous studies have reported short-term changes in psychological variables when stimulation was turned off or on. For instance, Berney and colleagues showed that depression, anxiety and elation remained stable after acute changes in the stimulation condition [63]. In contrast, Funkiewiez and colleagues [64] reported that both stimulation and medication were able to improve subjective feelings of well-being, euphoria and motivation, and to decrease anxiety and fatigue. Similarly, Amanzio and collaborators [23] showed that lower scores of depression and anxiety were reported by patients when psychological questionnaires were administered on medication as compared to their off medication state. Although we did not use questionnaires specifically designed for the administration in the short time (such as the Addiction Research Center Inventory, ARCI, [65] that was used in [64]), we explicitly asked patients to report how they felt in that particular condition of stimulation, thus ensuring that the scores really reflected their feelings in the on and off stimulation states. In addition, even if the BIS questionnaire asks to rate behaviours retrospectively and therefore is not specifically tailored to test the present state of mind, previous studies have shown that its scores may be affected by different medication conditions [66], [67], thus suggesting that the perception of impulsiveness may be affected by the therapy. The patients recruited for this study did not stop taking non-dopaminergic therapy, which has been shown to modulate impulsive behaviours [33]. However, it is unlikely that such medications biased the results as we varied only the stimulation state between conditions, thus differences in the results are highly likely to be explained by this latter factor.

In conclusion, we provide evidence that not all impulsivities are detrimentally affected by DBS of the STN and that the combined use of experimental paradigms and psychological questionnaires may be a helpful tool in reaching a convergent validity in the evaluation of cognitive changes in PD patients. Future studies are needed to further clarify how different subcomponents of impulsivity can be modulated both by dopaminergic drugs and STN-DBS. This issue is of great importance also from a clinical standpoint, in order to understand better and reduce the risk of post-operative suicidal behavior observed in some studies [68], possibly related not only to depression but also to impulsive behaviour. Altogether these findings contribute to a more general understanding of the role that STN-DBS has in treating behavioural abnormalities [69], [70]. Future multicenter studies on this clinically relevant issue, including larger cohorts of patients, will allow a more precise generalization of the results.
